# Contamination of Hospital Surfaces with Bacterial Pathogens under the Current COVID-19 Outbreak

**DOI:** 10.3390/ijerph18179042

**Published:** 2021-08-27

**Authors:** Andrei A. Pochtovyi, Daria V. Vasina, Daria D. Kustova, Elizaveta V. Divisenko, Nadezhda A. Kuznetsova, Olga A. Burgasova, Ludmila V. Kolobukhina, Artem P. Tkachuk, Vladimir A. Gushchin, Alexander L. Gintsburg

**Affiliations:** 1 Federal State Budget Institution “National Research Centre for Epidemiology and Microbiology Named after Honorary Academician N F Gamaleya” of the Ministry of Health of the Russian Federation, 123098 Moscow, Russia; d.v.vasina@gmail.com (D.V.V.); elizaveta.divisenko@yandex.ru (E.V.D.); nadyakuznetsova0@gmail.com (N.A.K.); lkolobuchina@yandex.ru (L.V.K.); artem.p.tkachuk@gmail.com (A.P.T.); gintsburg@gamaleya.org (A.L.G.); 2 Department of Virology, Biological Faculty, Lomonosov Moscow State University, 119991 Moscow, Russia; kustovad70@gmail.com; 3 Department of Infectious Diseases, Peoples’ Friendship University of Russia (RUDN University), 117198 Moscow, Russia; olgaburgasova@mail.ru; 4 Department of Infectiology and Virology, Federal State Autonomous Educational Institution of Higher Education I M Sechenov, First Moscow State Medical University of the Ministry of Health of the Russian Federation (Sechenov University), 119435 Moscow, Russia

**Keywords:** surface contamination, hospital infection, microbiome, COVID-19

## Abstract

The SARS-CoV-2 pandemic remains a global health issue for several reasons, such as the low vaccination rates and a lack of developed herd immunity to the evolution of SARS-CoV-2, as well as its potential inclination to elude neutralizing antibodies. It should be noted that the severity of the COVID-19 disease is significantly affected by the presence of co-infections. Comorbid conditions are caused not only by pathogenic and opportunistic microorganisms but also by some representatives of the environmental microbiome. The presence of patients with moderate and severe forms of the disease in hospitals indicates the need for epidemiological monitoring of (1) bacterial pathogens circulating in hospitals, especially the ESKAPE group pathogens, and (2) the microbiome of various surfaces in hospitals. In our study, we used combined methods based on PCR and NGS sequencing, which are widely used for epidemiological monitoring. Through this approach, we identified the DNA of pathogenic bacteria (*Klebsiella pneumoniae*, *Pseudomonas aeruginosa*, *Staphylococcus aureus*, CoNS, and *Achromobacter* spp.) on various surfaces. We also estimated the microbiome diversity of surfaces and identified the potential reservoirs of infections using 16S rRNA profiling. Although we did not assess the viability of identified microorganisms, our results indicate the possible risks of insufficient regular disinfection of surfaces, regardless of department, at the Infectious Diseases Hospital. Controlling the transmission of nosocomial diseases is critical to the successful treatment of COVID-19 patients, the rational use of antimicrobial drugs, and timely decontamination measures.

## 1. Introduction

The pandemic caused by severe acute respiratory syndrome (SARS-CoV-2) continues until now due to various factors such as the lack of developed herd immunity, the evolution of SARS-CoV-2, accompanied by the growing concerns about its potential ability to escape neutralizing antibodies [1,2,3], as well as the untimely partial/complete removal of restrictive measures. A year and a half following the first recorded case of COVID-19 infection, the world’s scientific and medical community has come to a better understanding of the main features and properties of the virus, and there is no longer any doubt about the mechanisms of virus spread through airborne droplets, as well as through fomites [4,5,6,7,8,9,10,11,12].

The primary SARS-CoV-2 infection is often accompanied by the occurrence of various comorbid conditions in patients, leading to various complications. These complications lead to a worsened course of the disease, increased duration of the patient’s stay in the hospital, and increased probability of a fatal outcome [13]. One of the triggers that causes a comorbid state is a coinfection. The causative agents of coinfection can be presented by widespread and rare microorganisms of bacterial, viral, and other etiological origins. Among them, bacteria are among the most frequent microorganisms in coinfection [14,15]. At the same time, the origin, distribution, and frequency of bacterial coinfection in patients with COVID-19 diagnosis have not yet been sufficiently studied. According to researchers, the presence of bacterial infection is observed in 3.5–50% of patients with COVID-19 [16,17]. The causative agents of nosocomial infections, which can be acquired by patients upon admission to the hospital, are of particular concern. Coinfection with such pathogens as *Staphylococcus aureus*, *Klebsiella pneumoniae*, *Pseudomonas aeruginosa*, *Streptococcus pneumoniae*, *Mycoplasma pneumoniae*, and *Acinetobacter baumannii* were mentioned in published studies [16,18,19,20]. Some of the listed microorganisms belong to the ESKAPE group of pathogens and are characterized by a high propensity to develop antibiotic resistance, which is of additional concern. Moreover, coinfections with conditionally pathogenic microorganisms have been noted, such as coagulase-negative staphylococci (CoNS) and *Stenotrophomonas maltophilia* [13,20], which also represent nosocomial pathogens with a substantial impact on human health. However, it is not entirely clear whether these are hospital-acquired or endogenous opportunist species.

In this regard, it is important to monitor the microbiological composition in a hospital setting to obtain up-to-date information about the epidemiological state and the necessity to take appropriate measures to prevent the spread of various infections, including nosocomial ones. Medical staff and patients are in close contact with different solid surfaces that are of particular interest as research objects due to the ability of microorganisms to be preserved on such surfaces for at least several days [21,22].

Methods based on polymerase chain reaction (PCR) and sequencing are widely used for epidemiological monitoring. PCR is a rapid method used to detect pathogenic microorganisms present in small quantities [23,24]. On the other hand, sequencing of 16S rRNA allows assessing a broader diversity of microbial populations [25]. Despite the limitations of 16S rRNA sequencing, the combined use of these two approaches in order to study the microbiome of medical institutions allows identifying common patterns in the distribution of microorganisms, as well as detecting potential reservoirs of nosocomial infections and developing new recommendations as preventive measures to reduce the risk of outbreaks of nosocomial infections.

Our research focused on the First Moscow Infectious Diseases Hospital, where we previously evaluated the contamination of RNA SARS-CoV-2 in the air and surfaces in various department [26]. Previously, we observed the highest aerosol contamination in the Intensive Care Unit (ICU) department (up to 513 copies per m^3^ of the air), while SARS-CoV-2 RNA was not detected in the aerosol in the Respiratory Infection Unit (RID). However, the surfaces were contaminated in both departments.

Thus, previous studies have been focused on the identification of the causative agent of COVID-19 [27], or identification of SARS-CoV-2, bacteria, and fungi (use cultural method) [28]. Here, we amend this omission, putting forward the main objectives of our study: (1) to identify pathogens of bacterial etiology circulating in hospitals and (2) to carry out a microbiome analysis of various surfaces in the ICU department and RID.

## 2. Materials and Methods

### 2.1. Sampling and Transportation

The study was carried out in the First Moscow Infectious Diseases Hospital (Russia), one of the capital’s medical institutions designated for the treatment of patients with COVID-19. During the first wave of COVID-19, about 80 patients with moderate to severe courses of the disease were admitted every day. Depending on the severity of the disease, the patients were assigned to the Respiratory Infections Department (RID) or to the Intensive Care Unit (ICU). Visitors’ access to patients was completely restricted, regardless of the department. Samples were collected in isolation rooms and hospital wards in RID and ICU from patients over the age of 18 with a positive PCR result and a confirmed diagnosis of COVID-19. Surface samples collected in the ICU department included swabs from the floor, door handles, and artificial lung ventilation apparatus screens. In the RID, the swabs were collected from the bedside tables, toilet seats, switches, window handles, floor, and door handles.

Doctors and medical staff collected nasopharyngeal swabs from hospitalized patients to detect SARS-CoV-2 RNA on admission. Nasopharyngeal swabs were transferred to a test tube containing 0.5 mL of sterile PBS solution. These probes were used for the qPCR analysis of pathogens in patients. Samples from various surfaces were collected 2–3 days after hospitalization of patients both in their close proximity and in public areas (hallway, first-aid post, and staff room). This time interval is optimal for possible contamination of various surfaces by patients [29]. Surface samples were collected using a sterile viscose swab (Tampon-probe, MiniMed, Northridge, Russia). Before collection, the swab was premoistened in sterile PBS solution, and samples were collected from a surface area of 25 cm^2^. The volume of each swab sample from the surface was 0.5 mL. All collected samples were immediately placed in a thermo bag at +4 °C and transported within 1–2 h to the laboratory with BSL-3. Obtained samples were aliquoted and deposited for preservation at −80 °C.

### 2.2. Nucleic Acid Extraction

DNA was extracted using a DNeasy PowerSoil Kit (Qiagen, Hilden, Germany) according to the manufacturer’s instructions. Pure swabs, extraction reagents, and a sterile PBS solution were used as negative controls.

### 2.3. qPCR for Bacterial Pathogen Identification

The identification and the quantification of DNA of methicillin-sensitive and methicillin-resistant *Staphylococcus aureus*, as well as methicillin-resistant coagulase-negative *Staphylococcus* spp., were performed using the «AmpliSens^®^ MRSA-screen-titer-FL» reagent kit (FSB of the Central Research Institute of Epidemiology of Rospotrebnadzor, Moscow, Russia) according to the manufacturer’s instructions. The detection of *Achromobacter* spp., *Burkholderia cepacia* complex, *Pseudomonas aeruginosa*, and *Klebsiella pneumonia* was carried out as previously described (Table S1, Supplementary Materials) [30,31,32,33].

### 2.4. 16S rRNA Gene Amplicon Sequencing

The hypervariable V4 region of the bacterial 16S rRNA gene was amplified using the forward primer 515F, 5′–GTGCCAGCMGCCGCGGTAA–3′ and the reverse primer 806R, 5′–GGACTACHVGGGTWTCTAAT–3′ [34]. Libraries were prepared using the NEBNext^®^ Fast DNA Library Prep Set for Ion Torrent™ (New England Biolabs, Ipswich, MA, USA) and barcoded with the use of Ion Code™ Barcode Adapters (Thermo Fisher Scientific, Waltham, MA, USA) according to the manufacturer’s instructions. DNA sequencing was performed using the Ion S5™ XL System (Thermo Fisher Scientific, Waltham, MA, USA). The sequence data were deposited in the NCBI Sequence Read Archive under accession number PRJNA737285.

### 2.5. Sequence Analysis

The analysis of the demultiplexed sequences was performed using the R package DADA2 (version 1.18.0) [35]. Filtering was performed using the filterAndTrim function; reads were truncated to 250 bp and cut from the 5′ end to 15 nucleotides. The maximum number of expected errors per read was set to 2. Denoising was performed taking into account the specifics of the Ion Torrent sequencing technology (single reads and Ion Torrent error recognition) using the following parameters: HOMOPOLYMER_GAP_PENALTY = −1, BAND_SIZE = 32. Chimeras were removed by the “consensus” method. The taxonomy assignment was performed using the R package DADA2 with the naïve Bayesian classifier method using the SILVA SSU v.132 [36] as a reference database of sequences. The ASV (taxonomy) table and metadata were imported for analysis using the R package phyloseq (version 1.34.0) [37]. Reads that were not assigned to the phylum level were removed before rarefaction. Rarefaction curves were evaluated before rarefying all samples to a common read depth of 41,140, which removed only two samples. Alpha-diversity analysis was performed using Chao1 and Shannon index. The Bray–Curtis dissimilarity matrix was ordered using PCoA. PERMANOVA tests were performed using the adonis2 function in the R package vegan (version 2.5.7) for samples pertaining to different departments and descriptions (surface type).

To estimate the most important features (genera) for the correct assignment of samples, a random forest analysis (R package randomForest, version 4.6.14) with the construction of 10,001 trees, using the genera as a classifier, was performed. All ASVs were merged to the genus level. Before performing normalization using Z-score, ASVs which had a prevalence <20% and a total abundance <50 were removed. The most important genera for the correct assignment of samples to departments were detected using the average reduction in model accuracy (MDA).

### 2.6. Ethical Consideration

The study was approved by local the Ethics Committee of the First Moscow Infectious Diseases Hospital, Moscow Department of Health, Moscow, Russia (Protocol No. 2/B, date of approval: 20 May 2020). Written informed consent was obtained from all participants.

## 3. Results

### 3.1. Detection of Bacterial Pathogens on Various Surfaces

Surface swabs were obtained from two departments of the First Moscow Infectious Diseases Hospital in general-purpose areas (hallway, staffrooms), in wards (in the RID), in the hallway, and in infectious rooms/anterooms (in the case of the ICU). A total of 13 and 22 surface swabs samples were collected in the ICU department and RID, respectively. Out of those samples, 13 were taken from the floor surface, eight were taken from the door handles, and three were taken from the surfaces of electronic devices. Nasopharyngeal swabs were collected from five and six patients in the ICU department and RID, respectively.

All samples were evaluated for the presence of methicillin-sensitive and methicillin-resistant *Staphylococcus aureus*, methicillin-resistant CoNS, *Achromobacter* spp., *Burkholderia cepacia* complex, *Pseudomonas aeruginosa*, and *Klebsiella pneumoniae*. PCR analysis estimated that all surface samples were contaminated with at least one pathogen from the test list (Table 1).

The most widespread microorganisms were CoNS (*n* = 35), which were found in all samples, regardless of the collection site. *Klebsiella pneumoniae* was identified in 100% of ICU samples and in 64% of RID samples. Thus, these two species were the main contaminants under the studied conditions. In addition, *Achromobacter* spp. (23%), *Staphylococcus aureus* (15%), and *Pseudomonas aeruginosa* (8%) were detected in the ICU. For RID, the distribution was slightly different, with *Pseudomonas aeruginosa* identified in about one-third of the samples (27%) and *Achromobacter* spp. identified in 14% of samples, while no *Staphylococcus aureus* was detected. *Burkholderia cepacia* complex was not found in any of the samples. The most contaminated surfaces were the floor (100%) and door handles (100%) (Table S2, Supplementary Materials).

### 3.2. Taxonomy Composition of Departments and Surface Types

Strict parameters of filtration quality and elimination of chimeric sequences were applied to microbiome data. After all filtering steps and elimination of low-quality reads, as well as chimeric sequences, 2,389,555 classified sequences were obtained and assigned to 8873 amplicon sequence variants (ASV) related to 990 genera and 357 families. All samples have reached a saturation point regarding α-diversity at a depth of 41,140 reads.

**Figure 1:**
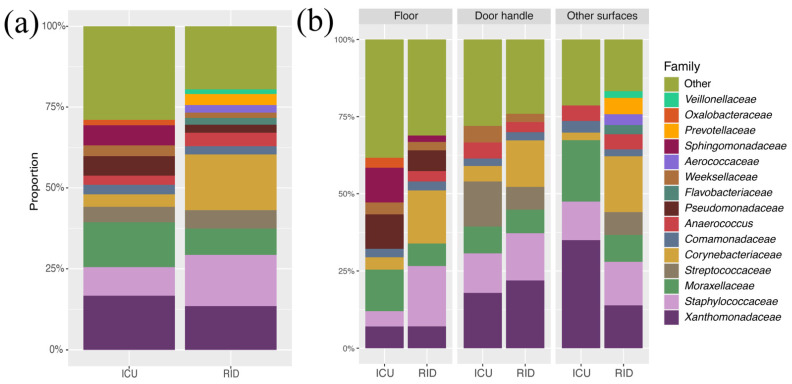
Relative taxonomic distribution at the family level: (**a**) by department; (**b**) by department and surface type. Families with a proportion of <2% are listed as “Other”. ICU—Intensive Care Unit; RID—Respiratory Infections Department.

The taxonomic distribution was identical between the two departments at the family level (Figure 1a). The first eight most frequent families were almost identical for the mentioned departments. The main differences in the 15 most widespread families were Weeksellaceae, Sphingomonadaceae, and Oxalobacteraceae for the ICU department, whereas, for the RID, there were Flavobacteriaceae, Aerococcaceae, and Prevotellaceae families. We also identified distinctions in the dominance of the most common families depending on the surface type (Figure 1b and Figure S1, Supplementary Materials).

### 3.3. Diversity

**Figure 2:**
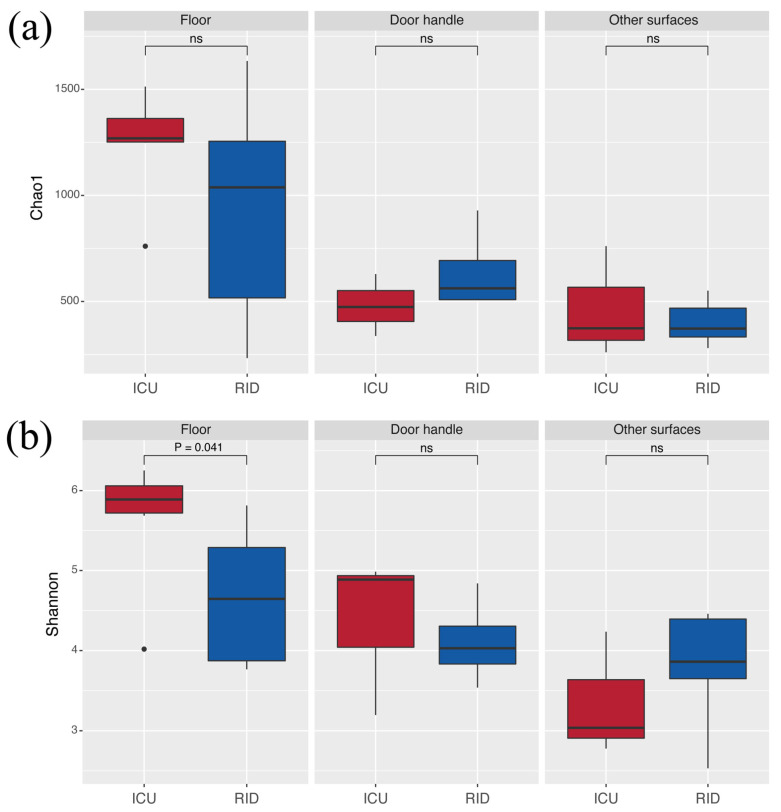
Dependence of α-diversity on the department and surface type: (**a**) diversity measured by the Chao1 index; (**b**) diversity measured by the Shannon index. ICU—Intensive Care Unit; RID—Respiratory Infections Department. Box plots with middle line denote the median, the box denotes the interquartile range (IQR), and 1.5 IQR ranges (whiskers). ns—no significance detected.

The alpha-diversity did not differ significantly between the samples collected in the ICU department and RID regarding the Chao1 and Shannon index (excluding floor surface comparison, *p* = 0.041) (Figure 2, Table S2 and Figure S2, Supplementary Materials).

According to the observed diversity, it is worth noting that Chao1 was higher for the floor surface regardless of the department, and the mean ± standard deviation value was 1234.7 ± 254.22 and 939.6 ± 547.46 for the ICU and RID, respectively. This pattern was maintained for the Shannon index, with the mean ± standard deviation of 5.64 ± 0.82 (ICU department) and 4.66 ± 0.87 (RID). It was approximately at the same level for door handles and other types of surfaces (swabs from sinks, toilet seats, and bedside table surfaces). Overall, the PCR results were reproduced, and the samples from the floor surface showed better diversity.

**Figure 3:**
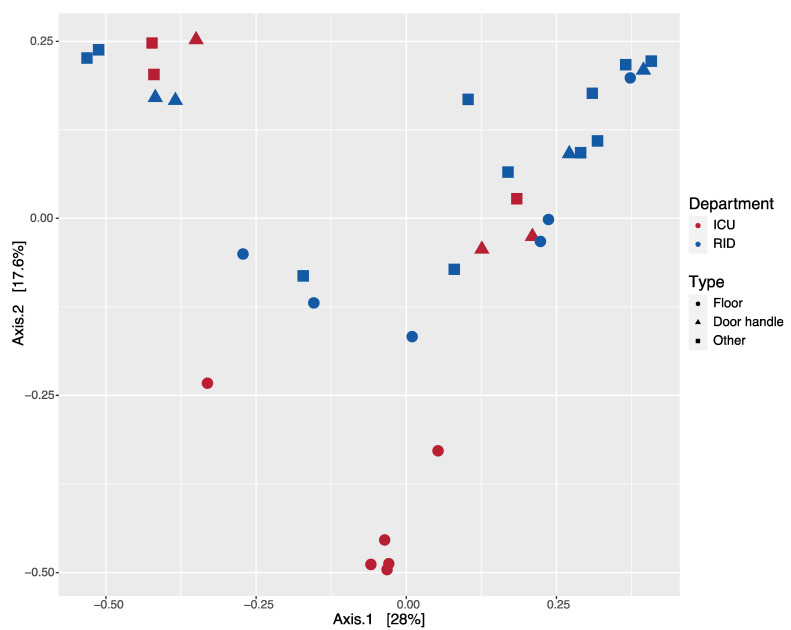
Bray–Curtis dissimilarity PCoA was used to generate ordination of beta-diversity in two departments. Principal coordinates 1 and 2 (Axis.1 and Axis.2) explained 28% and 17.6% of the variance in Bray–Curtis dissimilarity, respectively. Samples are colored according to the department (ICU—Intensive Care Unit; RID—Respiratory Infections Department), symbols indicate the type of sample.

We performed beta-diversity analysis by using PCoA based on Bray–Curtis dissimilarities. There were demonstrated both common and unique clusters for each department (Figure 3 and Figure S3, Supplementary Materials).

For the samples obtained from the floor in the ICU, we observed the formation of a separate cluster, indicating the high diversity of these samples. Other samples were evenly distributed throughout. We found significant differences in the multivariate PERMANOVA model with predictors such as department and description (surface type) (F = 3.48, R^2^ = 0.091, *p* = 0.0015 and F = 2.24, R^2^ = 0.125, *p* = 0.0036, respectively; Table S3, Supplementary Materials).

### 3.4. Search for Microbial Indicators of Departments

**Figure 4:**
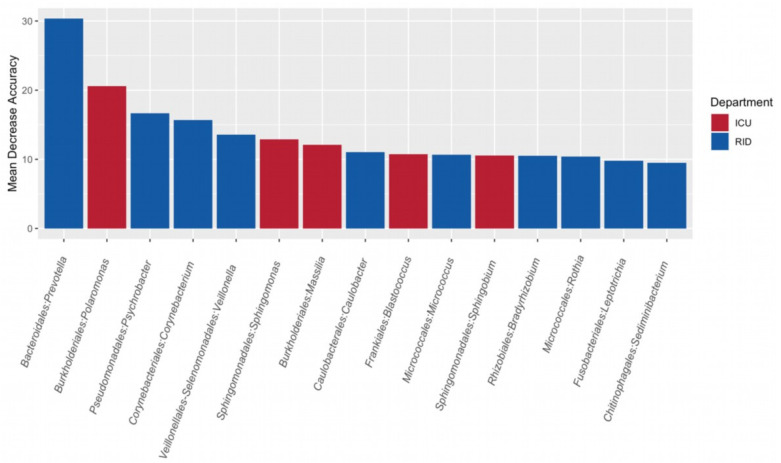
Random forest classification analysis of ICU (*n* = 12) and RID (*n* = 21) samples, showing taxonomic features with the highest classification variable importance for correctly identifying the department. ICU—Intensive Care Unit; RID—Respiratory Infections Department.

We used random forest and genera as classification features for department identification. This model had an out-of-bag error rate of 25.71%, with a substantial class error (42.86%) estimated for six ICU samples mistakenly assigned to RID. This may have been due to the large number of samples involved in the creation of such a model (21 RID samples versus 14 ICU samples). *Prevotella*, *Polaromonas*, *Psychrobacter*, and *Corynebacterium* were the most important genera for the precise classification of departments (MDA: 30.349, 20.570, 16.659, and 15.686, respectively; Figure 4 and Table S4, Supplementary Materials).

## 4. Discussion

The presence of coinfection among COVID-19 patients has been demonstrated in a growing number of studies. The conducted meta-analyses showed extremely heterogeneous data on the number of coinfections, reaching up to 50% of cases [16]. At the same time, nosocomial infections play a significant role in the formation of coinfection, influencing the course of the disease and increasing mortality [18].

Many recommendations for coronavirus infection treatment include the use of antibacterial drugs as a preventative measure against bacterial infections [38,39,40]. This certainly raises concerns about the overuse of antibiotics and the emergence of multidrug-resistant bacteria, which is already a global public health problem. Monitoring is required for bacterial pathogen identification given the high chances of transmission of bacterial infections in hospital settings and the necessity for rational use of antibiotics. This information will allow eliminating the reservoirs of infections and promptly preventing outbreaks of nosocomial infections.

Hospital surfaces are often contaminated with various microorganisms and can be potential reservoirs for the spread of microbial agents [22]. In this regard, we studied various surfaces in the Infectious Diseases Hospital in Moscow. The choice of surfaces for our study was determined by the characteristics of each department. Patients in critical condition were admitted to the ICU department. Given the immobility of these patients, our attention was focused on surfaces such as the floor, door handles, and artificial lung ventilation apparatus screens, i.e., the main surfaces that the medical staff comes into contact with on a daily basis. On the other hand, in the RID, patients can move around inside their wards and visit the bathrooms. In this regard, we expanded the list of studied surfaces and included bedside tables, toilet seats, switches, window handles, etc. (Table S2 Supplementary Materials).

In our study, all the surfaces were contaminated with at least one pathogen, regardless of the department. CoNS and *Klebsiella pneumoniae* were the most frequently detected pathogens and were found in almost every surface swab test (for CoNS in all samples). In general, coagulase-negative staphylococcus represents the normal human skin flora and is less pathogenic than *Staphylococcus aureus* [41]. However, cases of CoNS bloodstream infections (BSI) and catheter-related bloodstream infections (CRBSI) have been reported among patients with COVID-19 [13]. At the same time, *Klebsiella pneumoniae* has been associated with several nosocomial outbreaks and occurs in patients alongside new coronavirus infection [18,29,42,43].

A search for pathogens such as *Achromobacter* spp. and *Burkholderia cepacia* complex was also conducted. These microorganisms are usually identified among people with weakened immune systems and patients with cystic fibrosis. Recently, these microorganisms have been noted as etiological agents that can cause pneumonia [44,45]. Importantly, only *Burkholderia cepacia* complex was found in the respiratory tract of patients with COVID-19 [46,47].

The microbiome study allowed us to confirm the assumptions regarding the diversity of bacterial composition on the floor surface and door handle. The results of qPCR and microbiome sequencing data were consistent.

*Polaromonas*, *Sphingomonas*, and *Massilia* genera were the most characteristic for the ICU department, while *Prevotella*, *Psychrobacter*, *Corynebacterium*, and *Veillonella* genera were the most characteristic for the RID. The genera data can be considered as a “marker” for department identification. It is worth noting that these genera are representative of both a normal human microflora and an ordinary environment. However, some representatives from this list have been found in the bloodstream of COVID-19 patients (*Sphingomonas* [48]), whereas representatives of *Prevotella* were more common in the upper respiratory tract among patients with SARS-CoV-2 infection and *Corynebacterium* was represented among healthy patients [49]. Oropharyngeal microbiome analysis of patients with COVID-19 demonstrated high levels of *Veillonella* [50].

In addition, *Staphylococcus aureus* and *Pseudomonas aeruginosa* were identified on the door handles and floor surface. Moreover, *Pseudomonas aeruginosa* was found on the sink mixer located in the ward. In previous studies, contamination with these microorganisms on door handles was also demonstrated. It is worth noting that the detection rate of such pathogens was higher in studies similar to ours (more than 6%) [22,51]. However, given the pathogenic potential of these microorganisms, their detection in the ICU department is of particular concern. The results of our study on *Klebsiella pneumoniae*, *Pseudomonas pneumoniae*, and *Staphylococcus aureus* spread are consistent with previously published data, despite the peculiarities of health systems in different countries [52,53,54,55]. These microorganisms are inclined toward biofilm formation and possess other pathogenicity factors [29], which increase the risk of infection in patients with coronavirus infection.

More importantly, the nasopharyngeal smears of patients admitted to the hospital did not contain above-mentioned pathogens; therefore, we consider the patients themselves an unlikely source of contamination for the surrounding surfaces. Apparently, the spread of pathogens may be enhanced by medical staff, whereas it may also be associated with low cleaning efficiency and contamination by previously hospitalized patients.

Taking the obtained results into account, these surfaces (floor and door handles) can be considered as potential reservoirs of nosocomial infections that increase the risk of infection spread both inside and outside the hospital. The results indicate the danger of insufficient regular disinfection of surfaces, regardless of department, at the Infectious Diseases Hospital.

## 5. Conclusions

In this study, we demonstrated a combined approach to characterize the microbiome of different surfaces for the presence of pathogens that could induce comorbid conditions in patients with COVID-19. Epidemiological monitoring is extremely important for preventing the outbreaks of disease in a hospital setting as well as for the rational use of antimicrobial drugs and timely implementation of decontamination measures. This will improve the epidemiological situation and improve the quality of medical care.

## Figures and Tables

**Table 1:** Number of positive detections in surface samples and in nasopharyngeal swabs from patients by PCR.

Bacterial Pathogen	Intensive Care Unit		Respiratory Infections Department	
	Surface (*n* = 13)	Patient (*n* = 5)	Surface (*n* = 22)	Patient (*n* = 6)
*Klebsiella pneumoniae*	13 (100%)	0 (0%)	14 (63.64%)	0 (0%)
*Pseudomonas aeruginosa*	1 (7.69%)	0 (0%)	6 (27.27%)	0 (0%)
*Staphylococcus aureus*	2 (15.38%)	0 (0%)	0 (0%)	0 (0%)
CoNS	13 (100%)	0 (0%)	22 (100%)	0 (0%)
*Achromobacter* spp.	3 (23.08%)	0 (0%)	3 (13.64%)	0 (0%)
*Burkholderia cepacia* complex	0 (0%)	0 (0%)	0 (0%)	0 (0%)

## Data Availability

The sequence data have been deposited in the NCBI Sequence Read Archive under accession number PRJNA737285.

## Supplementary Materials

The following are available online at https://www.mdpi.com/article/10.3390/ijerph18179042/s1, Table S1: List of oligonucleotide primers used in this study. Table S2: Swab collection points from various surfaces. Table S3: PERMANOVA model, with predictors department and description explaining 21.5% of among-sample diversity (Bray–Curtis dissimilarity). Table S4: Random forest classification models of department. Figure S1: Relative taxonomic distribution at the family level for all surface types. Families with a proportion of <2% are listed as “Other”. Figure S2: Dependence of α-diversity on the department and surface types: (a) diversity measured by the Chao1 index; (b) diversity measured by the Shannon index. Figure S3: Ordination of beta-diversity in two departments and surface types. Bray–Curtis dissimilarity PCoA was used to characterize the diversity. Samples are colored according to the type of surface; symbol indicates the department.

## References

[1] Chen X., Chen Z., Azman A.S., Sun R., Lu W., Zheng N., Zhou J., Wu Q., Deng X., Zhao Z. (2021). Neutralizing antibodies against SARS-CoV-2 variants induced by natural infection or vaccination: A systematic review and pooled meta-analysis. Clin. Infect. Dis..

[2] Harvey W.T., Carabelli A.M., Jackson B., Gupta R.K., Thomson E.C., Harrison E.M., Ludden C., Reeve R., Rambaut A., Peacock S.J. (2021). SARS-CoV-2 variants, spike mutations and immune escape. Nat. Rev. Microbiol..

[3] Alenquer M., Ferreira F., Lousa D., Valério M., Medina-Lopes M., Bergman M.-L., Gonçalves J., Demengeot J., Leite R.B., Lilue J. (2021). Signatures in SARS-CoV-2 spike protein conferring escape to neutralizing antibodies. PLoS Pathog..

[4] Ghinai I., McPherson T.D., Hunter J.C., Kirking H.L., Christiansen D., Joshi K., Rubin R., Morales-Estrada S., Black S.R., Pacilli M. (2020). First known person-to-person transmission of severe acute respiratory syndrome coronavirus 2 (SARS-CoV-2) in the USA. Lancet.

[5] Pastorino B., Touret F., Gilles M., de Lamballerie X., Charrel R.N. (2020). Prolonged Infectivity of SARS-CoV-2 in Fomites. Emerg. Infect. Dis..

[6] Peng X., Xu X., Li Y., Cheng L., Zhou X., Ren B. (2020). Transmission routes of 2019-nCoV and controls in dental practice. Int. J. Oral Sci..

[7] Richard M., Kok A., de Meulder D., Bestebroer T.M., Lamers M.M., Okba N.M.A., Fentener van Vlissingen M., Rockx B., Haagmans B.L., Koopmans M.P.G. (2020). SARS-CoV-2 is transmitted via contact and via the air between ferrets. Nat. Commun..

[8] Ong S.W.X., Tan Y.K., Chia P.Y., Lee T.H., Ng O.T., Wong M.S.Y., Marimuthu K. (2020). Air, Surface Environmental, and Personal Protective Equipment Contamination by Severe Acute Respiratory Syndrome Coronavirus 2 (SARS-CoV-2) from a Symptomatic Patient. J. Am. Med. Assoc..

[9] Wang Y., Qiao F., Zhou F., Yuan Y. (2020). Aerosol and Surface Distribution of Severe Acute Respiratory Syndrome Coronavirus 2 in Hospital Wards, Wuhan, China, 2020. Indoor Built Environ..

[10] Nissen K., Krambrich J., Akaberi D., Hoffman T., Ling J., Lundkvist Å., Svensson L., Salaneck E. (2020). Long-distance airborne dispersal of SARS-CoV-2 in COVID-19 wards. Sci. Rep..

[11] Santarpia J.L., Rivera D.N., Herrera V.L., Morwitzer M.J., Creager H.M., Santarpia G.W., Crown K.K., Brett-Major D.M., Schnaubelt E.R., Broadhurst M.J. (2020). Aerosol and surface contamination of SARS-CoV-2 observed in quarantine and isolation care. Sci. Rep..

[12] Liu Y., Ning Z., Chen Y., Guo M., Liu Y., Gali N.K., Sun L., Duan Y., Cai J., Westerdahl D. (2020). Aerodynamic analysis of SARS-CoV-2 in two Wuhan hospitals. Nature.

[13] Bardi T., Pintado V., Gomez-rojo M., Escudero-sanchez R., Lopez A.A. (2021). Nosocomial infections associated to COVID-19 in the intensive care unit: Clinical characteristics and outcome. Eur. J. Clin. Microbiol. Infect. Dis..

[14] Li Z., Chen Z.M., Chen L.D., Zhan Y.Q., Li S.Q., Cheng J., Zhu A.R., Chen L.Y., Zhong N.S., Li S.Y. (2020). Coinfection with SARS-CoV-2 and other respiratory pathogens in patients with COVID-19 in Guangzhou, China. J. Med. Virol..

[15] He S., Liu W., Jiang M., Huang P., Xiang Z., Deng D., Chen P., Xie L. (2021). Clinical characteristics of COVID-19 patients with clinically diagnosed bacterial co-infection: A multi-center study. PLoS ONE.

[16] Langford B.J., So M., Raybardhan S., Leung V., Westwood D., MacFadden D.R., Soucy J.P.R., Daneman N. (2020). Bacterial co-infection and secondary infection in patients with COVID-19: A living rapid review and meta-analysis. Clin. Microbiol. Infect..

[17] Lai C.C., Wang C.Y., Hsueh P.R. (2020). Co-infections among patients with COVID-19: The need for combination therapy with non-anti-SARS-CoV-2 agents?. J. Microbiol. Immunol. Infect..

[18] Arcari G., Raponi G., Sacco F., Bibbolino G., Di Lella F.M., Alessandri F., Coletti M., Trancassini M., Deales A., Pugliese F. (2021). *Klebsiella pneumoniae* infections in COVID-19 patients: A 2-month retrospective analysis in an Italian hospital. Int. J. Antimicrob. Agents.

[19] Cusumano J.A., Dupper A.C., Malik Y., Gavioli E.M., Banga J., Berbel Caban A., Nadkarni D., Obla A., Vasa C.V., Mazo D. (2020). *Staphylococcus aureus* Bacteremia in Patients Infected with COVID-19: A Case Series. Open Forum Infect. Dis..

[20] Rusic D., Vilovic M., Bukic J., Leskur D., Seselja Perisin A., Kumric M., Martinovic D., Petric A., Modun D., Bozic J. (2021). Implications of COVID-19 pandemic on the emergence of antimicrobial resistance: Adjusting the response to future outbreaks. Life.

[21] Kramer A., Schwebke I., Kampf G. (2006). How long do nosocomial pathogens persist on inanimate surfaces? A systematic review. BMC Infect. Dis..

[22] Bhatta D.R., Hamal D., Shrestha R., Hosuru Subramanya S., Baral N., Singh R.K., Nayak N., Gokhale S. (2018). Bacterial contamination of frequently touched objects in a tertiary care hospital of Pokhara, Nepal: How safe are our hands?. Antimicrob. Resist. Infect. Control.

[23] van Beek J., de Graaf M., Al-Hello H., Allen D.J., Ambert-Balay K., Botteldoorn N., Brytting M., Buesa J., Cabrerizo M., Chan M. (2018). Molecular surveillance of norovirus, 2005–2016: An epidemiological analysis of data collected from the NoroNet network. Lancet Infect. Dis..

[24] Desdouits M., de Graaf M., Strubbia S., Oude Munnink B.B., Kroneman A., Le Guyader F.S., Koopmans M.P.G. (2020). Novel opportunities for NGS-based one health surveillance of foodborne viruses. One Health Outlook.

[25] Rampelotto P.H., Sereia A.F.R., De Oliveira L.F.V., Margis R. (2019). Exploring the hospital microbiome by high-resolution 16s rRNA profiling. Int. J. Mol. Sci..

[26] Pochtovyi A.A., Bacalin V.V., Kuznetsova N.A., Nikiforova M.A., Shidlovskaya E.V., Verdiev B.I., Milashenko E.N., Shchetinin A.M., Burgasova O.A., Kolobukhina L.V. (2021). SARS-CoV-2 Aerosol and Surface Contamination in Health Care Settings: The Moscow Pilot Study. Aerosol Air Qual. Res..

[27] Chia P.Y., Coleman K.K., Tan Y.K., Ong S.W.X., Gum M., Lau S.K., Lim X.F., Lim A.S., Sutjipto S., Lee P.H. (2020). Detection of air and surface contamination by SARS-CoV-2 in hospital rooms of infected patients. Nat. Commun..

[28] Hemati S., Mobini G.R., Heidari M., Rahmani F., Soleymani Babadi A., Farhadkhani M., Nourmoradi H., Raeisi A., Ahmadi A., Khodabakhshi A. (2021). Simultaneous monitoring of SARS-CoV-2, bacteria, and fungi in indoor air of hospital: A study on Hajar Hospital in Shahrekord, Iran. Environ. Sci. Pollut. Res..

[29] Hassan M.Z., Sturm-Ramirez K., Rahman M.Z., Hossain K., Aleem M.A., Bhuiyan M.U., Islam M.M., Rahman M., Gurley E.S. (2019). Contamination of hospital surfaces with respiratory pathogens in Bangladesh. PLoS ONE.

[30] Price E.P., Arango V.S., Kidd T.J., Fraser T.A., Nguyen T.K., Bell S.C., Sarovich D.S. (2020). Duplex real-time PCR assay for the simultaneous detection of *Achromobacter xylosoxidans* and *Achromobacter* spp.. Microb. Genom..

[31] Deschaght P., De Baere T., Van Simaey L., Van Daele S., De Baets F., De Vos D., Pirnay J.P., Vaneechoutte M. (2009). Comparison of the sensitivity of culture, PCR and quantitative real-time PCR for the detection of *Pseudomonas aeruginosa* in sputum of cystic fibrosis patients. BMC Microbiol..

[32] Martinucci M., Roscetto E., Iula V.D., Votsi A., Catania M.R., De Gregorio E. (2016). Accurate identification of members of the *Burkholderia cepacia* complex in cystic fibrosis sputum. Lett. Appl. Microbiol..

[33] Hartman L.J., Selby E.B., Whitehouse C.A., Coyne S.R., Jaissle J.G., Twenhafel N.A., Burke R.L., Kulesh D.A. (2009). Rapid real-time PCR assays for detection of *Klebsiella pneumoniae* with the rmpA or magA genes associated with the hypermucoviscosity phenotype: Screening of nonhuman primates. J. Mol. Diagn..

[34] Caporaso J.G., Lauber C.L., Walters W.A., Berg-Lyons D., Lozupone C.A., Turnbaugh P.J., Fierer N., Knight R. (2011). Global patterns of 16S rRNA diversity at a depth of millions of sequences per sample. Proc. Natl. Acad. Sci. USA.

[35] Davis N.M., Proctor D.M., Holmes S.P., Relman D.A., Callahan B.J. (2018). Simple statistical identification and removal of contaminant sequences in marker-gene and metagenomics data. Microbiome.

[36] Quast C., Pruesse E., Yilmaz P., Gerken J., Schweer T., Yarza P., Peplies J., Glöckner F.O. (2013). The SILVA ribosomal RNA gene database project: Improved data processing and web-based tools. Nucleic Acids Res..

[37] McMurdie P.J., Holmes S. (2013). Phyloseq: An R Package for Reproducible Interactive Analysis and Graphics of Microbiome Census Data. PLoS ONE.

[38] Wang D., Hu B., Hu C., Zhu F., Liu X., Zhang J., Wang B., Xiang H., Cheng Z., Xiong Y. (2020). Clinical Characteristics of 138 Hospitalized Patients with 2019 Novel Coronavirus-Infected Pneumonia in Wuhan, China. J. Am. Med. Assoc..

[39] Lucien M.A.B., Canarie M.F., Kilgore P.E., Jean-Denis G., Fénélon N., Pierre M., Cerpa M., Joseph G.A., Maki G., Zervos M.J. (2021). Antibiotics and antimicrobial resistance in the COVID-19 era: Perspective from resource-limited settings. Int. J. Infect. Dis..

[40] Langford B.J., So M., Raybardhan S., Leung V., Soucy J.P.R., Westwood D., Daneman N., MacFadden D.R. (2021). Antibiotic prescribing in patients with COVID-19: Rapid review and meta-analysis. Clin. Microbiol. Infect..

[41] Becker K., Heilmann C., Peters G. (2014). Coagulase-negative staphylococci. Clin. Microbiol. Rev..

[42] Peña C., Pujol M., Ardanuy C., Ricart A., Pallarés R., Liñares J., Ariza J., Gudiol F. (2001). An outbreak of hospital-acquired *Klebsiella pneumoniae* bacteraemia, including strains producing extended-spectrum β-lactamase. J. Hosp. Infect..

[43] Hosoda T., Harada S., Okamoto K., Ishino S., Kaneko M., Suzuki M., Ito R., Mizoguchi M. (2021). COVID-19 and fatal sepsis caused by hypervirulent *Klebsiella pneumoniae*, Japan, 2020. Emerg. Infect. Dis..

[44] Choudhury S., Papineni S., Ramachandruni S., Molina J., Surani S. (2021). *Achromobacter xylosoxidans*/denitrificans Bacteremia in a Patient with Good’s Syndrome. Cureus.

[45] Awadh H., Mansour M., Aqtash O., Shweihat Y. (2017). Pneumonia due to a Rare Pathogen: *Achromobacter xylosoxidans*, Subspecies denitrificans. Case Rep. Infect. Dis..

[46] Osman H., Nguyen P. (2020). First Case of COVID-19 Complicated with Burkolderia Cepacia Pneumonia and Bacteremia. Chest.

[47] Zhong H., Wang Y., Shi Z., Zhang L., Ren H., He W., Zhang Z., Zhu A., Zhao J., Xiao F. (2021). Characterization of respiratory microbial dysbiosis in hospitalized COVID-19 patients. Cell Discov..

[48] Sirivongrangson P., Kulvichit W., Payungporn S., Pisitkun T., Chindamporn A., Peerapornratana S., Pisitkun P., Chitcharoen S., Sawaswong V., Worasilchai N. (2020). Endotoxemia and circulating bacteriome in severe COVID-19 patients. Intensive Care Med. Exp..

[49] Rosas-Salazar C., Kimura K.S., Shilts M.H., Strickland B.A., Freeman M.H., Wessinger B.C., Gupta V., Brown H.M., Rajagopala S.V., Turner J.H. (2021). SARS-CoV-2 infection and viral load are associated with the upper respiratory tract microbiome. J. Allergy Clin. Immunol..

[50] Ma S., Zhang F., Zhou F., Li H., Ge W., Gan R., Nie H., Li B., Wang Y., Wu M. (2021). Metagenomic analysis reveals oropharyngeal microbiota alterations in patients with COVID-19. Signal Transduct. Target. Ther..

[51] Oie S., Hosokawa I., Kamiya A. (2002). Contamination of room door handles by methicillin-sensitive/methicillin-resistant *Staphylococcus aureus*. J. Hosp. Infect..

[52] Caneiras C., Lito L., Melo-Cristino J., Duarte A. (2019). Community-and hospital-acquired *Klebsiella pneumoniae* urinary tract infections in Portugal: Virulence and antibiotic resistance. Microorganisms.

[53] Engelhart S.T., Krizek L., Glasmacher A., Fischnaller E., Marklein G., Exner M. (2002). *Pseudomonas aeruginosa* outbreak in a haematology-oncology unit associated with contaminated surface cleaning equipment. J. Hosp. Infect..

[54] Nkuwi E.J., Kabanangi F., Joachim A., Rugarabamu S., Majigo M. (2018). Methicillin-resistant *Staphylococcus aureus* contamination and distribution in patient’s care environment at Muhimbili National Hospital, Dar es Salaam-Tanzania. BMC Res. Notes.

[55] De Abreu P.M., Farias P.G., Paiva G.S., Almeida A.M., Morais P.V. (2014). Persistence of microbial communities including *Pseudomonas aeruginosa* in a hospital environment: A potential health hazard. BMC Microbiol..

