# Supplementary material for: Contamination of Hospital Surfaces with Bacterial Pathogens under the Current COVID-19 Outbreak
Source: Int J Environ Res Public Health. 2021 Aug 27;18(17):9042. doi: 10.3390/ijerph18179042 (PMC8431522; doi:10.3390/ijerph18179042)
Supplement: Supplementary file 1 [file ijerph-18-09042-s001.zip › ijerph-1282395-supplementary.pdf]

Table S1. List of oligonucleotide primers used in this study.

Bacterial pathogen	Target	Sequence	Sources
Achromobacter spp.	rpoB<br>(DNA-directed RNA polymerase subunit beta)	Ac_F 5'-CACrTAGCTCACGAACTCCAAGC-3'<br>Ac_R 5'-CAGCTTCAATCCTACCTAACTTTCCT-3'<br>Ac_probe 5'-HEX-CGTAGCCGACGGTTTGCAGG-BHQ1-3'	[1]
Burkholderia cepacia complex			
Burkholderia cenocepacia	Bcal_1709<br>(TonB-dependent siderophore receptor)	Fw1 5'-GACTGGGAATTGCGCCATAC-3'<br>Rv1 5'-CTTCGCGACGTAGCTTTGC-3'<br>P1 5'-FAM-TTGCGCAGCACCTTCGACAATACCT-BHQ1-3'	
Burkholderia ambifaria	Bamb_1889<br>(isochorismatase hydrolase)	Fw1 5'-TGTCGGAGATCAAAGGGTTGT-3'<br>Rv1 5'-CGTGCACGCGTCGAAA-3'<br>P1 5'-JOE-CTCGGACATCGTGGCCAAGGACC-BHQ1-3'	
Burkholderia vietnamiensis	Bcep1808_1697<br>(2-nitropropane dioxygenase)	Fw1 5'-CTCGATCGATACCTGCGTAAC-3'<br>Rv1 5'-AGTGCCCGAACTTCTCGAT-3'<br>P1 5'-CY5-GCGCATCCGCAGCAAGCTC-BHQ2-3'	
Burkholderia gladioli	bgla_1 g00140<br>(LuxR family DNA-binding response regulator)	Fw1 5'-GCAAGATCGACGAGGTGGAT-3'<br>Rv1 5'-GTAATTCGCGCCGGCATA-3'<br>P1 5'-CY5-ATCTGATCGCCGCCACGCATG-BHQ2-3'	
Burkholderia multivorans	Bmul_5209<br>(hypothetical protein)	Fw1 5'-GCGGCGAGGSAAAGAAG-3'<br>Rv1 5'-CGCTGGCGGAATATTTGAAG-3'<br>Rv2 5'-CGCTGGCGGAATATTTGAAG-3'<br>P1 5'-FAM-CCTTTTCCGGTATCGACGACGCTCTC-BHQ1-3'<br>P2 5'-FAM-CCTTTTCCAATATCGAGGACGCTCTC-BHQ1-3'	[2]
Burkholderia cepacian	GEM_2222<br>(gluconate 2-dehydrogenase)	Fw1 5'-GCGTTGCTGGCCAAACC-3'<br>Rv1 5'-TTGCACACGCGTCACATG-3'<br>P1 5'-FAM-AATTTCGAGTTGCGCGACCGCTC-BHQ1-3'	
	GEM_5741<br>(membrane protein)	Fw1 5'-CGCGATCCATTTCGGAAT-3'<br>Fw1 5'-TGCGCTCCATTTCGTGTT-3'<br>Rv1 5'-AGCAGGATTGCCTCGATCAG-3'<br>Rv2 5'-AACAGGATCGCCTCGACCAG-3'<br>P1 5'-FAM-CGTGCTGCTGWCGCGGTACTGG-BHQ1-3'	
Burkholderia dolosa	AK34_3542<br>(hypothetical protein)	Fw1 5'-ACATGCTGCAGCTGATTGAC-3'<br>Rv1 5'-TGCAACGTACCAAACGTCAG-3'<br>P1 5'-CY5-AAAGCGCTGGATGACAACGCTTG-BHQ2-3'	
Burkholderia pyrrocinia	ABD05_16660<br>(hypothetical protein)	Fw1 5'-GTTTCATCTCCTCGGCTTCC-3'<br>Rv1 5'-ACATCGAGGAAACGAAGCTG-3'<br>P1 5'-JOE-ATGGAACCTGTTTCCGCGAGTGAG-BHQ1-3'	

Pseudomonas aeruginosa	oprL<br>(outer membrane protein)	PAO1 Sa 5'-ACC CGA ACG CAG GCT ATG-TET-3'<br>PAO1 Aa 5'-CAG GTC GGA GCT GTC GTA CTC-3'<br>oprL TMc 5'-FAM-AGAAGGTGGTGATCGCACGCAGA-BHQ1-3'	[3]
Klebsiella pneumonia	Khe<br>(Hemolysin)	F 5'-GATGAAACGACCTGATTGCATTC-3'<br>R 5'-CCGGGCTGTCGGGATAAG-3'<br>P 5'-FAM-CGCGAACTGGAAGGGCCCG-BHQ1-3'	[4]

References

1. Price, E.P.; Arango, V.S.; Kidd, T.J.; Fraser, T.A.; Nguyen, T.K.; Bell, S.C.; Sarovich, D.S. Duplex real-time PCR assay for the simultaneous detection of Achromobacter xylosoxidans and Achromobacter spp. *Microb. Genomics* **2020**, *6*, 1–11, doi:10.1099/mgen.0.000406.
2. Martinucci, M.; Roscetto, E.; Iula, V.D.; Votsi, A.; Catania, M.R.; De Gregorio, E. Accurate identification of members of the Burkholderia cepacia complex in cystic fibrosis sputum. *Lett. Appl. Microbiol.* **2016**, *62*, 221–229, doi:10.1111/lam.12537.
3. Deschaght, P.; De Baere, T.; Van Simaey, L.; Van Daele, S.; De Baets, F.; De Vos, D.; Pirnay, J.P.; Vaneechoutte, M. Comparison of the sensitivity of culture, PCR and quantitative real-time PCR for the detection of Pseudomonas aeruginosa in sputum of cystic fibrosis patients. *BMC Microbiol.* **2009**, *9*, 1–7, doi:10.1186/1471-2180-9-244.
4. Hartman, L.J.; Selby, E.B.; Whitehouse, C.A.; Coyne, S.R.; Jaissle, J.G.; Twenhafel, N.A.; Burke, R.L.; Kulesh, D.A. Rapid real-time PCR assays for detection of Klebsiella pneumoniae with the rmpA or magA genes associated with the hypermucoviscosity phenotype: Screening of nonhuman primates. *J. Mol. Diagnostics* **2009**, *11*, 464–471, doi:10.2353/jmoldx.2009.080136.

Table S2. Swab collection points from various surfaces. ICU – Intensive Care Unit; RID - Respiratory Infections Department.

Point	Department	Zone	Location	Data	Description	Chao1	Shannon	Cycle threshold (Ct)							
								SASR-CoV-2	*Achromobacter* spp.	*Burkholderia cepacia complex*	*Pseudomonas aeruginosa*	*Klebsiella pneumoniae*	*Staphylococcus-CoNS*	*Staphylococcus aureus*	Methicillin resistance mecA
A1	ICU	Clean	Entrance to the department, Hallway	25.06.2020	Floor	1372.861	5.96	neg	32.182	neg	neg	32.364	28.277	neg	29.958
A2	ICU	Clean	Entrance to the department, Hallway	25.06.2020	Door handle	622.333	4.884	neg	neg	neg	neg	32.576	27.763	33.730	29.356
A5	ICU	Clean	Senior nurses room	25.06.2020	Door handle	473.333	4.98	neg	neg	neg	neg	32.539	29.318	Neg	33.026
A9	ICU	Clean	Staff room	25.06.2020	Floor	1258.513	5.822	neg	31.656	neg	32.071	33.550	25.640	31.434	27.956
A11	ICU	Red	Room №49, 2 patients	25.06.2020	Floor	1249.313	5.689	pos	neg	neg	neg	31.468	27.467	Neg	28.999
A12	ICU	Red	Room №49, 2 patients	25.06.2020	Electronics screen	375.923	2.772	pos	neg	neg	neg	34.704	28.388	neg	30.075
A15	ICU	Red	Anteroom №49	25.06.2020	Door handle	337.647	3.192	pos	neg	neg	neg	30.482	27.402	neg	30.138
A17	ICU	Red	Room №52, 2 patients	25.06.2020	Floor	1497.88	6.092	pos	neg	neg	neg	29.943	28.760	neg	30.592
A23	ICU	Red	Room №54, 1 patient	25.06.2020	Floor	Sample was removed after rarefaction		neg	neg	neg	neg	33.414	28.881	neg	30.128
A24	ICU	Red	Room №54, 1 patient	25.06.2020	Electronics screen	762.077	4.225	neg	neg	neg	neg	34.792	28.077	neg	29.877
A35	ICU	Red	Room №58, 1 patient	25.06.2020	Floor	760.905	4.029	neg	neg	neg	neg	31.763	28.468	neg	31.177
A36	ICU	Red	Room №58, 1 patient	25.06.2020	Electronics screen	260.071	3.039	neg	neg	neg	neg	30.837	29.895	neg	32.778
A45	ICU	Red	Anteroom №58	25.06.2020	Floor	1268.429	6.256	pos	31.606	neg	neg	31.597	25.949	neg	28.072
2	RID	-	Patient room №14, 2 patients	15.06.2020	Floor	369.526	4.165	pos	neg	neg	31.325	neg	22.956	neg	23.971
3	RID	-	Patient room №14, 2 patients	15.06.2020	Toilet seat	Sample was removed after rarefaction		neg	neg	neg	neg	neg	25.668	neg	35.306
4	RID	-	Patient room №14, 2 patients	15.06.2020	Sink (tap)	396.667	4.023	neg	neg	neg	neg	neg	30.937	neg	32.323
5	RID	-	Patient room №14, 2 patients	15.06.2020	Bedside table	366.885	3.687	pos	neg	neg	neg	neg	27.426	neg	27.633
6	RID	-	Patient room №14, 2 patients	15.06.2020	Window handle	387.556	3.836	pos	neg	neg	neg	35.001	24.525	neg	26.22
7	RID	-	Patient room №14, 2 patients	15.06.2020	Door handle	496.6	4.124	neg	neg	neg	neg	neg	25.401	neg	26.816
9	RID	-	Patient room №14, 2 patients	15.06.2020	Switch	370.333	4.408	neg	neg	neg	neg	neg	24.813	neg	26.998
12	RID	-	Patient room №14, 2 patients	18.06.2020	Floor	234	3.767	neg	28.734	neg	21.204	28.271	24.699	neg	26.984

13	RID	-	Patient room №14, 2 patients	18.06.2020	Toilet seat	302	4.46	neg	neg	neg	neg	30.375	25.911	neg	27.108
15	RID	-	Patient room № 17, 4 patients	18.06.2020	Floor	963.788	5.352	neg	31.511	neg	25.102	31.584	24.684	neg	27.208
30	RID	-	Patient room № 17, 4 patients	18.06.2020	Phone screen	535.459	4.451	neg	neg	neg	neg	25.186	27.664	neg	32.041
42	RID	-	Patient room № 17, 4 patients	18.06.2020	Call button	297.276	2.751	neg	neg	neg	neg	35.555	29.371	neg	33.078
51	RID	-	Patient room № 17, 4 patients	18.06.2020	Air conditioning	400.885	2.541	neg	neg	neg	neg	36.810	31.701	neg	33.301
54	RID	-	Patient room № 17, 4 patients	18.06.2020	Sink (tap)	282.04	3.86	neg	neg	neg	34.188	31.711	23.896	neg	29.806
56	RID	-	Patient room № 17, 4 patients	18.06.2020	Door handle	512.457	3.558	pos	neg	neg	neg	31.390	27.917	neg	29.376
57	RID	-	Patient room №18, 1 patient	15.06.2020	Switch	547.667	4.384	neg	neg	neg	neg	30.530	28.865	neg	29.981
61	RID	-	Patient room №18, 1 patient	15.06.2020	Bedside table	553.143	3.633	neg	neg	neg	neg	34.237	26.869	neg	27.980
64	RID	-	Staff room	15.06.2020	Door handle	926.448	4.825	neg	neg	neg	neg	32.124	26.313	neg	27.851
68	RID	-	Staff room	16.06.2020	Floor	1293.577	3.774	neg	neg	neg	neg	neg	23.727	neg	28.297
72	RID	-	Treatment room	16.06.2020	Floor	1649.881	5.806	neg	neg	neg	neg	neg	27.371	neg	28.929
78	RID	-	Sanitary room	18.06.2020	Floor	1126.759	5.119	neg	31.940	neg	31.000	31.660	23.700	neg	23.940
81	RID	-	Sanitary room	18.06.2020	Door handle	623.887	3.934	neg	neg	neg	32.715	27.733	26.809	neg	28.396
K- (Swab)	-	-	-	-	-	-	-	neg	neg	neg	neg	neg	neg	neg	neg
K- (PBS)	-	-	-	-	-	-	-	neg	neg	neg	neg	neg	neg	neg	neg

Table S3. PERMANOVA model, with predictors department and description explaining 21.5% of among-sample diversity (Bray–Curtis dissimilarity).

Predictor	Df	Sum Sq.	R^2	F	*p*-value
Department	1	0.9009	0.09061	3.4787	0.0015
Description (Surface type)	2	1.2454	0.12525	2.4045	0.0036
Department:Description (Surface type)	2	0.8046	0.08091	1.5533	0.0655
Residual	27	6.9924	0.70323		
Total	32	9.9433	1.00000		

Table S4. Random forest classification models of department. MDAs (Mean decrease in accuracy) are given for the 15 most important genera for classifying samples

Department: Intensive Care Unit/Respiratory Infections Department			
Out-of-bag estimate of error rate: 25.71%			
Confusion matrix	Intensive Care Unit	Respiratory Infections Department	Class error (%)
Intensive Care Unit	8	6	42.86
Respiratory Infections Department	3	18	14.29
Most important genera in sample classification			
Class:Genera	Intensive Care Unit	Respiratory Infections Department	MDA
Bacteroidales:Prevotella	26.826	25.605	30.349
Burkholderiales:Polaromonas	15.871	18.894	20.570
Pseudomonadales:Psychrobacter	12.431	15.911	16.659
Corynebacteriales:Corynebacterium	14.596	11.181	15.686
Veillonellales-Selenomonadales:Veillonella	10.556	11.281	13.560
Sphingomonadales:Sphingomonas	8.335	13.235	12.871
Burkholderiales:Massilia	9.293	11.144	12.102
Caulobacterales:Caulobacter	8.473	10.514	11.040
Frankiales:Blastococcus	5.105	10.784	10.724
Micrococcales:Micrococcus	6.504	10.189	10.644
Sphingomonadales:Sphingobium	3.592	11.277	10.534
Rhizobiales:Bradyrhizobium	6.754	10.444	10.513
Micrococcales:Rothia	6.945	9.628	10.401
Fusobacteriales:Leptotrichia	7.210	8.359	9.806
Chitinophagales:Sediminibacterium	10.064	5.037	9.483

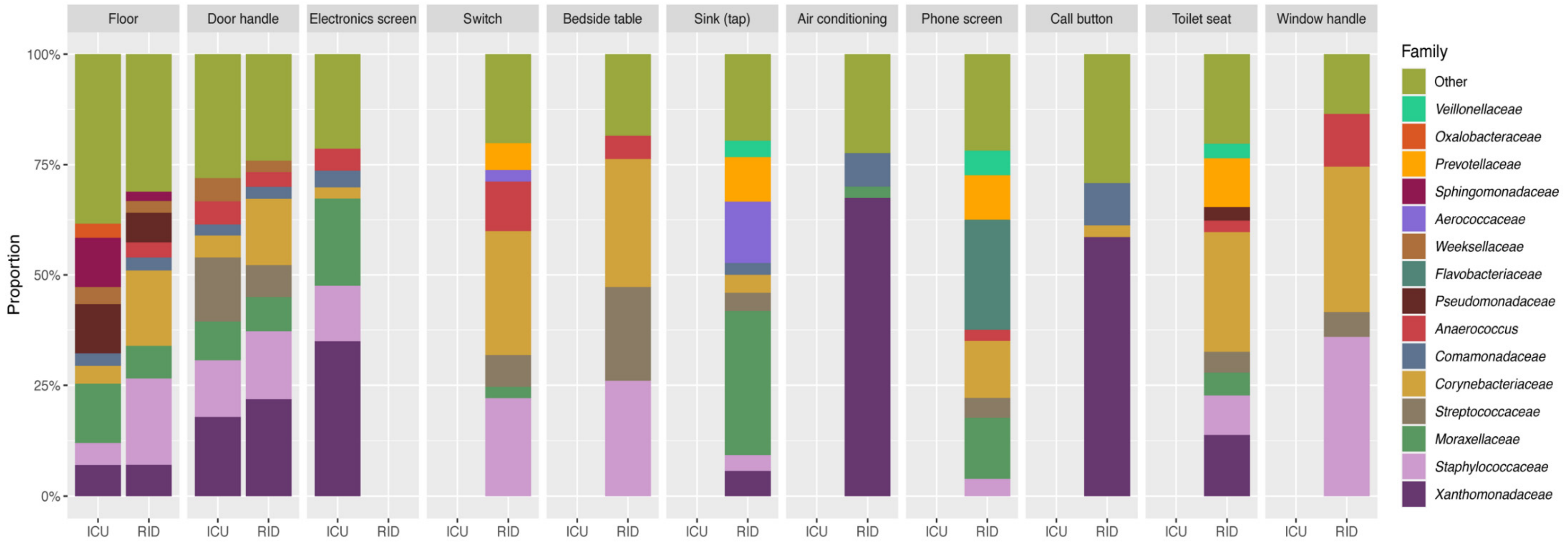

Figure S1. Relative taxonomic distribution at the family level for all surface types. Families with a proportion of <2% are listed as "Other".

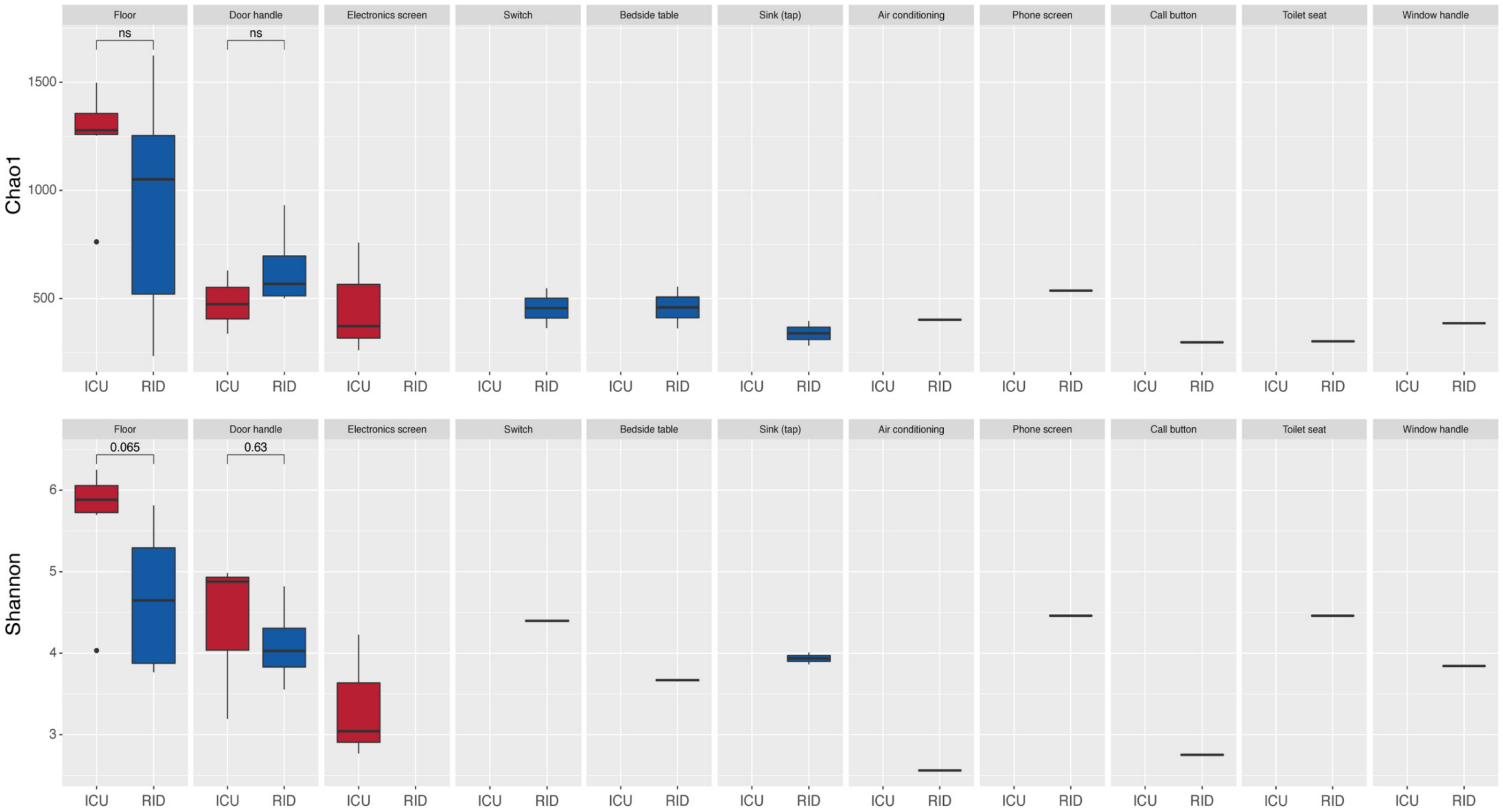

Figure S2. Dependence of α-diversity on the department and surface types: (a) diversity measured by the Chao1 index; (b) diversity measured by the Shannon index.

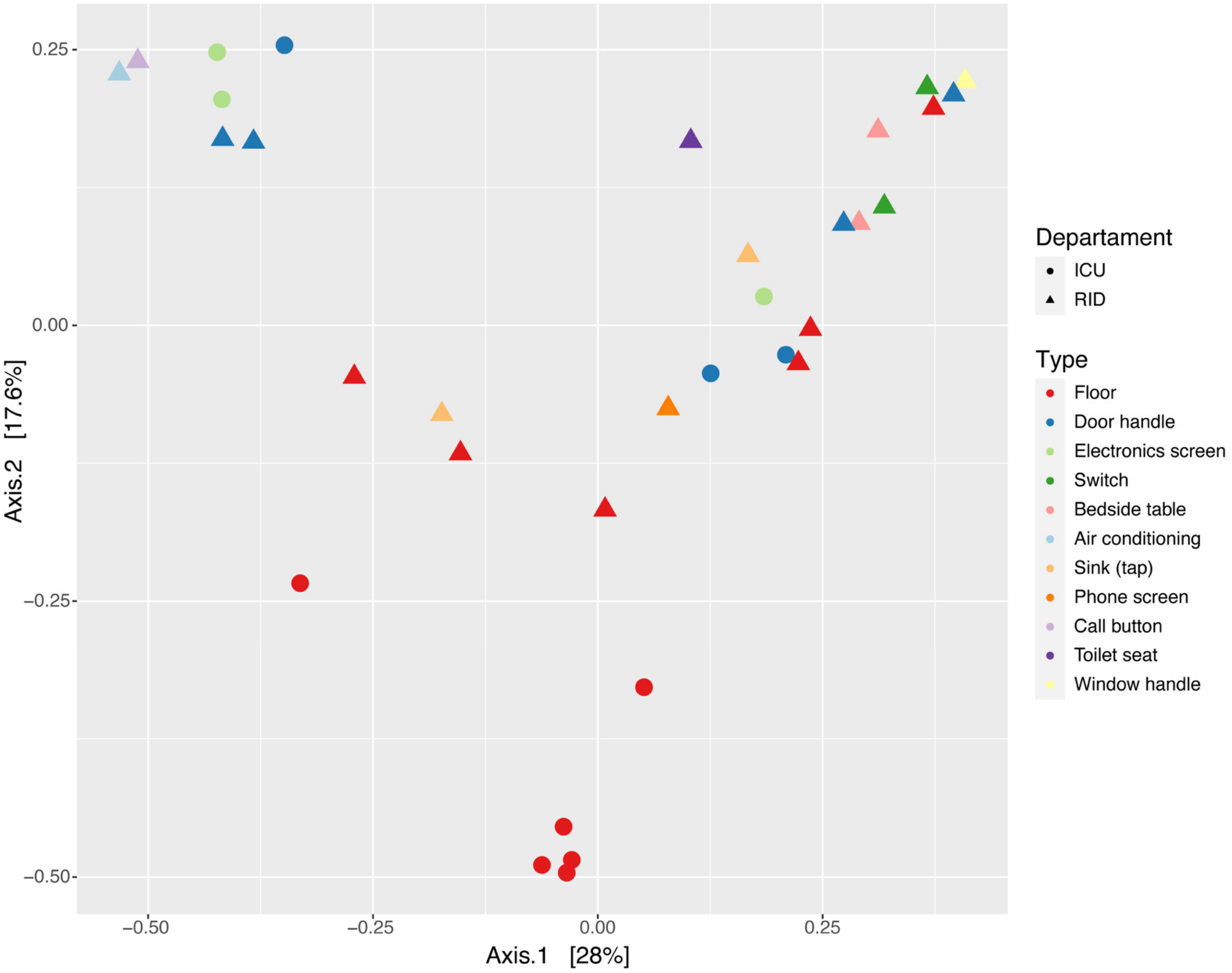

Figure S3. Ordination of beta-diversity in two departments and surface types. Bray–Curtis dissimilarity PCoA was used to characterize the diversity. Samples are colored according to the type of surface; symbol indicates the department.
